# Direct Composite Restorations on Permanent Teeth in the Anterior and Posterior Region – An Evidence-Based Clinical Practice Guideline – Part 2: Recommendations for Composite Processing

**DOI:** 10.3290/j.jad.b5749192

**Published:** 2024-09-17

**Authors:** Caroline Sekundo, Cornelia Frese, Roland Frankenberger, Rainer Haak, Andreas Braun, Norbert Krämer, Gabriel Krastl, Falk Schwendicke, Esra Kosan, Eva Langowski, Diana Wolff, and Guideline Panel Members and Methodological Consultants

**Affiliations:** a Dentist, Department of Conservative Dentistry, Heidelberg University, University Hospital Heidelberg, Germany. Project administration, methodology, investigation, writing (original draft).; b Adjunct Professor, Department of Conservative Dentistry, Heidelberg University, University Hospital Heidelberg, Germany. Project administration, investigation, writing (review and editing).; c Professor, Department of Operative Dentistry and Endodontology, University of Marburg, Germany. Project administration, investigation, writing (review and editing).; d Professor, Department of Cariology, Endodontology and Periodontology, University of Leipzig, Germany. Project administration, investigation, writing (review and editing).; e Professor, Department of Operative Dentistry, Periodontology and Preventive Dentistry, RWTH Aachen University, Germany. Investigation, writing (review and editing).; f Professor, Paediatric Dentistry, University of Gießen, Germany. Investigation, writing (review and editing).; g Professor, Department of Conservative Dentistry and Periodontology, Center of Dental Traumatology, University Hospital of Würzburg, Germany. Investigation, writing (review and editing).; h Professor, Department of Conservative Dentistry and Periodontology, LMU Klinikum, Germany. Project administration, investigation, writing (review and editing).; i Dentist, Department of Periodontology, Oral Medicine and Oral Surgery, Charité – Universitätsmedizin Berlin, Germany. Methodology, investigation, writing (review and editing).; j Dentist, Department of Conservative Dentistry, Heidelberg University, University Hospital Heidelberg, Germany. Methodology, investigation, writing (review and editing).; k Professor, Department of Conservative Dentistry, Heidelberg University, University Hospital Heidelberg, Germany. Supervision, funding acquisition, project administration, investigation, writing (review and editing).

**Keywords:** caries removal, acid etching, adhesion, polymerization, adhesive restorations, composite resin, composite restorations

## Abstract

**Purpose:**

Part 2 of this German S3 clinical practice guideline provides recommendations for the process of manufacturing composite restorations. It covers key aspects like caries removal, field isolation, matrix and adhesive techniques, as well as light curing and polishing. The outcomes of interest include survival rates and restoration quality.

**Materials and Methods:**

A systematic literature search was conducted by two methodologists using MEDLINE and the Cochrane Library via the OVID platform, including studies up to December 2021. Additionally, the reference lists of relevant manuscripts were manually reviewed. Six PICO questions were developed to guide the search. Consensus-based recommendations were formulated by a panel of dental professionals from 20 national societies and organizations based on the collected evidence and expert opinion.

**Results:**

The guideline advocates for one-stage selective caries removal near the pulp and underscores the effectiveness of various isolation techniques, adhesive systems, and the crucial role of light polymerization. The use of anatomically preformed sectional matrices and phosphoric acid etching is recommended to enhance restoration quality. Additionally, polishing composite restorations is advised to improve surface finish.

**Conclusion:**

This guideline provides comprehensive recommendations that inform clinicians on optimizing the composite restoration manufacturing processes. The adoption of these best practices can improve the quality and longevity of dental restorations.

Composite restorations have emerged as a versatile solution in modern dentistry, providing durable and esthetically pleasing results for caries treatment.^41^ However, achieving successful outcomes requires adherence to correct manufacturing procedures that include crucial aspects like caries excavation, field isolation, matrix and adhesive techniques, light polymerization, and polishing.

Caries excavation forms the cornerstone of the restoration process. Two principal approaches are employed: non-selective excavation, which involves removing all carious tissue down to healthy tooth structure throughout the cavity, and selective excavation, which leaves caries-altered tissue near the pulp intact to minimize exposure.^4^ While this selective approach may affect the available bonding surface for the composite, it prioritizes pulp survival, offering a critical balance between effective excavation and tooth preservation. Furthermore, this method can sometimes be combined as part of a two-step removal of caries.^55^ Understanding the optimal balance between these methods is important to enhance the long-term success of composite restorations.

Following excavation, proper work field isolation is crucial for preventing contamination by moisture, bacteria, and debris, which could impair adhesion and esthetics. Effective contamination control ensures a secure, lasting bond between composite and tooth structure. Techniques such as rubber dam provide “absolute” isolation by physically separating the treatment area, while suction devices, cotton rolls, drying pastes, and air-drying systems are systems for “relative” work field isolation.^40,57^ Matrix systems also contribute significantly to contamination control and should be assessed for their efficiency in providing adequate isolation.

Moreover, matrix systems play a pivotal role in shaping and contouring the restoration. Depending on the clinical scenario, clinicians may choose from various materials and techniques. Plastic matrices, due to their transparency, facilitate light polymerization but are less stable than metal matrices, which provide greater rigidity. Anatomical matrices are designed to mimic natural tooth contours for precise shaping, whereas straight matrices are often simpler to apply. A circular matrix technique employs a thin band wrapped around the whole tooth, while the partial matrix technique focuses on covering only the proximal area with secure attachment through rings, wedges, or fluid rubber dam.^26^ Evaluating these various matrix systems and techniques is essential to identify the most effective approach for maintaining optimal contours and proximal contact points.

Adhesive systems underpin the success of composite restorations, ensuring a strong bond to tooth structure despite the inherent shrinkage of composites. These systems have evolved over time, from the traditional three-step and two-step etch-and-rinse strategies to two-step and one-step self-etch systems.56 Universal adhesives offer further flexibility across different clinical situations. While etch-and-rinse systems using phosphoric acid remain effective for enamel conditioning, unintentional dentin etching can occur as cavity size decreases. Self-etch systems simplify the process by omitting phosphoric acid, while hydrophobic bonding agents often found in three-step etch-and-rinse and two-step self-etch adhesives enhance dentin durability. Comparing these strategies is crucial for determining the adhesive protocols that optimize bonding and reduce clinical failures.^8^

Light polymerization is essential for curing composites, yet it often presents challenges due to handling errors and equipment limitations.^11^ Ensuring proper handling and reliable polymerization units is critical for consistent results. For bulk-fill composites, there is uncertainty about whether the manufacturers’ promised depth of cure can be achieved consistently. Investigating polymerization protocols and equipment requirements can clarify these uncertainties and contribute to more reliable light curing.

Lastly, polishing composite restorations enhances patient satisfaction by reducing surface roughness and minimizing plaque buildup.^2^ However, it remains unclear whether polishing truly prolongs clinical retention or minimizes secondary caries. Evaluating the impact of polishing on long-term clinical performance will offer valuable insights into the overall benefits of this practice.

Given the wide array of techniques outlined, part 2 of this guideline aims to provide action recommendations for process quality of the manufacturing process in terms of quality assurance. This guideline primarily targets dentists but also aims to offer additional information to patients and their caregivers.

## Methods

This guideline was created according to the methodological standards set by the Standing Guideline Commission of the Association of Scientific Medical Societies in Germany (AWMF). It was developed under the leadership of the German Society of Restorative Dentistry (DGZ) and the German Society of Dentistry and Oral Medicine (DGZMK). A guideline panel was assembled, consisting of dental professionals from 20 national societies and organizations to ensure comprehensive representation. For a list of all participating organizations, please see our publication on part 1 of the guideline.^62^ An Organizing Committee and a team of methodology consultants appointed by the DGZMK supervised the development process. Participants in the guideline development process were nominated, actively contributed to the work, and had voting rights during the consensus conference. The methodology consultants provided guidance to participants but did not hold voting rights. Therapeutic questions were identified and framed as Population, Intervention, Comparator, and Outcome (PICO) questions. The guideline panel prioritized the questions based on clinical relevance and feasibility within the project’s timeline. Part 2 of this guideline addresses PICO questions 6–11. The questions addressed can be found in Table 1. The target patient population consists of individuals with permanent tooth structure loss needing restoration, excluding those with endodontically pre-treated teeth, build-up restorations, structural anomalies like molar incisor hypomineralization, or those requiring complete bite elevation.

**Table 1:** PICO(S) questions

PICO question	6 Caries Excavation	7 Work field isolation	8 Matrix technique	9 Adhesive technique	10 Light polymerization	11 Finishing and polishing
PICO aspect	Explanation					
Population	Patients with permanent teeth and carious defects requiring treatment, insufficient restorations or hypersensitive teeth (without endodontically pre-treated teeth, build-up fillings, MIH or other structural anomalies)	Patients with permanent teeth and carious defects requiring treatment, insufficient restorations or hypersensitive teeth (without endodontically pre-treated teeth, build-up fillings, MIH or other structural anomalies)	Patients with permanent teeth and carious defects requiring treatment, insufficient restorations or hypersensitive teeth (without endodontically pre-treated teeth, build-up fillings, MIH or other structural anomalies)	Patients with permanent teeth and carious defects requiring treatment, insufficient restorations or hypersensitive teeth (without endodontically pre-treated teeth, build-up fillings, MIH or other structural anomalies)	Patients with permanent teeth and carious defects requiring treatment, insufficient restorations or hypersensitive teeth (without endodontically pre-treated teeth, build-up fillings, MIH or other structural anomalies)	Patients with permanent teeth and carious defects requiring treatment, insufficient restorations or hypersensitive teeth (without endodontically pre-treated teeth, build-up fillings, MIH or other structural anomalies)
Intervention	Non-selective caries excavation (conventional) in combination with composite restoration	Absolute work field isolation (rubber dam)	Acrylic matrix Teflon tape Sectional matrices Wedges	Etch and rinse technique, multi-bottle	Light curing of direct composite restorations	Finishing, polishing of direct composite restorations
Comparison control	Selective caries excavation, in combination with composite restoration	Relative work field isolation	Search without specifying comparison, selection during screening, e.g., matrix band	One-bottle, universal, selective etching or similar	–	–
Outcome	Survival rate	Survival rate	Survival rate	Survival rate	Survival rate	Survival rate Quality indicators, surface gloss/surface discoloration
Study type/setting	Study designs: Systematic reviews, meta-analyses At least 12 months’ follow-up At least 15 restorations Publication since 1990 Languages: German, English, French, Russian	Study designs: CCTs, RCTs Systematic reviews, meta-analyses At least 12 months’ follow-up At least 15 restorations Publication since 1990 Languages: German, English, French, Russian	Study designs: CCTs, RCTs Systematic reviews, meta-analyses Prospective/retrospective cohort studies At least 12 months’ follow-up At least 15 restorations Publication since 1990 Languages: German, English, French, Russian	Study designs: Systematic reviews, meta-analyses At least 12 months’ follow-up At least 15 restorations Publication since 1990 Languages: German, English, French, Russian	Study designs: CCTs, RCTs Prospective/retrospective cohort studies Systematic reviews, meta-analyses At least 12 months’ follow-up At least 15 restorations Publication since 1990 Languages: German, English, French, Russian	Study designs: CCTs, RCTs Systematic reviews, meta-analyses At least 12 months’ follow-up At least 15 restorations Publication since 1990 Languages: German, English, French, Russian

CCT= Controlled Clinical Trial. RCT = Randomized Clinical Trial.

A systematic search was conducted independently by two investigators (CS and EL) up to December 2021. Two electronic databases, the National Library of Medicine, Washington, DC. (MEDLINE via OVID), and the Cochrane Library (CENTRAL), were searched to address the research questions. Additionally, reference lists of relevant manuscripts were manually reviewed. Table A.1 in the Appendix provides details of the search strategies for PICO questions 6–11. General inclusion criteria required *in-vivo* studies that have a follow-up period of at least 12 months, include a minimum of 15 restorations examined, and be published in English, German, French, or Russian from 1990 onwards. The details of included populations and study designs varied based on each PICO question, as outlined in Table 1. Studies that did not fulfill all inclusion criteria were excluded.

For feasibility reasons, the systematic evaluation of evidence was limited to PICO questions 1–5 (see part 1)^62^. For PICO questions 6–11, concerning composite processing, a systematic literature search was conducted and relevant literature was then provided to the panel in February 2022. Consequently, these recommendations are consensus-based.

Based on the provided literature and expert opinion, the guideline’s recommendations were formulated by separate working groups in alignment with AWMF specifications. The recommendations from the working groups were made available to the guideline coordinator. The guideline document was then provided to the guideline group four weeks before the consensus conference. During the NIH Type 1 structured consensus conference,^25^ the recommendations were presented to the plenary session by the working group, and participants had the opportunity to ask questions or submit reasoned amendments. The recommendations and amendments were then voted on. If necessary, further discussions were held to develop alternative proposals, which were followed by a final vote.

Tables 2 and 3 outline the methods used to determine the strength of the recommendations and classify consensus levels.

**Table 2:** Strength of recommendations: grading scheme (German Association of the Scientific Medical Societies [AWMF] and Standing Guidelines Commission)^16^

	Recommendation	Recommendation against intervention	Description	Symbol
A	Shall/We recommend	Shall not/We do not recommend	Strong recommendation	↑↑ resp. ↓↓
B	Should/We propose	Should not/We do not suggest	Recommendation	↑ resp. ↓
0	Can/May be considered	Can be dispensed with	Open recommendation	↔

**Table 3:** Strength of consensus: determination scheme (German Association of the Scientific Medical Societies [AWMF] and Standing Guidelines Commission)^10^

Strong consensus	Agreement of >95% of participants
Consensus	Agreement of >75 to 95% of participants
Simple majority	Agreement of >50 to 75% of participants
No consensus	Agreement of <50% of the participants

## Results

**Fig 1:**
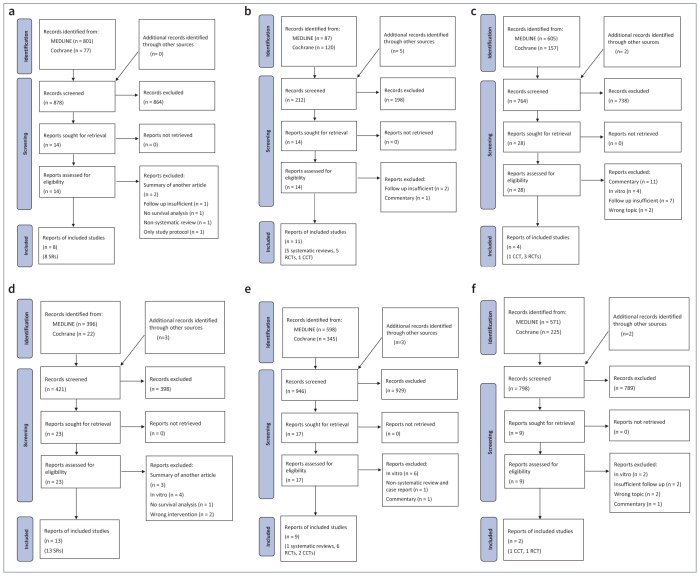
PRISMA Flow diagrams for the PICO questions. (a) PICO question #6, (b) PICO question #7, (c) PICO question #8, (d) PICO question #9, (e) PICO question #10, (f) PICO question #11.

Figure 1 displays the PRISMA (Preferred Reporting Items for Systematic Reviews and Meta-Analyses) flow diagrams used for literature selection. The Appendix (Table A.2) contains detailed lists of excluded manuscripts along with the reasoning for each PICO question. In the area of caries excavation, eight systematic reviews were identified.^4,10,22,27,32,52,54,55^ For contamination control, the search results included five systematic reviews^7,9,39,40,60^ and six clinical trials.^18,24,35,46,47,57^ Regarding matrix technique, only four *in-vivo* studies could be identified.^12,20,21,45^ Thirteen systematic reviews were found on adhesive techniques.^1,15,17,19,31,34,39,43,44,50,51,53,58^ For light polymerization, one systematic review 33 and 8 clinical trials were found.^3,5,13,14,23,30,59,61^ Two studies addressed polishing.^28,42^

All resulting recommendations and statements were agreed upon by strong consensus. In total, part 2 of the guideline resulted in seven consensus-based recommendations and two consensus-based statements. These are presented below (Tables 4–12).

### Caries Excavation

**Table 4:** Consensus-based recommendation 1

Both selective and non-selective caries excavation procedures **can** be used. In the case of dentin lesions close to the pulp, one-stage selective caries removal **should** be preferred to stepwise or non-selective caries removal. Vote: 16/0/0 (yes, no, abstention)	Strong consensus
Further reading: Barros et al., 2020,^4^ Hoefler et al., 2016,^27^ Li et al., 2018,^32^ Schwendicke et al., 2013,^52^ Schwendicke et al., 2013,^54^ Schwendicke et al., 2021^55^	

### Contamination Control/Work Field Isolation

**Table 5:** Consensus-based recommendation 2

Both relative and absolute isolation techniques **can** be successfully used to control contamination in direct composite restorations on permanent teeth. Contamination control with a rubber dam (absolute isolation) could have a positive effect on the longevity of the restorations in the long term. Vote: 16/0/0 (yes, no, abstention)	Strong consensus
Further reading: Brunthaler et al., 2003,^7^ Cajazeira et al., 2014,^9^ Daudt et al., 2013,^18^ Loguercio et al., 2015,^35^ Mahn et al., 2015,^39^ Miao et al., 2021,^40^ Raskin et al., 2000,^46^ Sabbagh et al., 2017,^47^ Smales et al., 1992,^57^ Wang et al., 2016^60^	

### Matrix Technique

**Table 6:** Consensus-based recommendation 3

Both metal and acrylic matrices **can** be used for sufficient proximal contact design. Vote: 16/0/0 (yes, no, abstention)	Strong consensus
Further reading: Cenci et al., 2007,^12^ Demarco et al., 2007,^20^ Demarco et al., 2010,^21^ Prakki et al., 2003^45^	

**Table 7:** Consensus-based recommendation 4

An anatomically preformed sectional matrix in combination with a wedge and ring system **should** be preferred for Class II restorations to optimize the contact point design and avoid excess. Vote: 16/0/0 (yes, no, abstention)	Strong consensus
Further reading: Kampouropoulos et al., 2010,^29^ Loomans et al., 2006,^36^ Loomans et al., 2008,^38^ Loomans et al., 2009,^37^ Saber et al., 2010,^49^ Saber et al., 2011^48^	

### Adhesive Technique

**Table 8:** Consensus-based recommendation 5

To improve the long-term quality of the enamel margin and prevent marginal discoloration, the enamel of all direct composite restorations** should** be etched with phosphoric acid. Vote: 16/0/0 (yes, no, abstention)	Strong consensus
Further reading: Askar et al., 2021,^1^ Krithikadatta et al., 2010,^31^ Mahn et al., 2015,39 Szesz et al., 2016^58^	

**Table 9:** Consensus-based recommendation 6

Two-step-self-etch, three-step-etch-and-rinse adhesive systems or universal adhesives** should** be preferred for direct composite restorations. Vote: 16/0/0 (yes, no, abstention)	Strong consensus
Further reading: Peumans et al., 2005,^44^ Schwendicke et al., 2016,^53^ De Assis et al., 2020^19^	

### Light Polymerization

**Table 10:** Consensus-based statement 1

Light polymerization is a decisive factor for the clinical success of composite restorations. The correct handling (eg, polymerization direction, distance, diameter of the light cone), the energy applied (power × time) and the opacity and shade of the composite are relevant. Vote: 17/0/0 (yes, no, abstention)	Strong consensus
Further reading: Lima et al., 2015,^33^ Cerruti et al., 2020^13^	

**Table 11:** Consensus-based statement 2

Bulk-fill composites **can** be polymerized safely up to a depth of 4 mm with polymerization units of appropriate power. Vote: 17/0/0 (yes, no, abstention)	Strong consensus
Further reading: Lima et al., 2015^33^	

### Polishing and Finishing

**Table 12:** Consensus-based statement 3

The composite restoration **should** be polished to improve the surface and reduce plaque build-up. Vote: 16/0/0 (yes, no, abstention)	Strong consensus
Further literature: Jung et al., 2005,^28^ Nassar et al., 2014^42^	

## Discussion

The development of this guidelines reflects a comprehensive analysis aimed at improving the quality and predictability of composite restorations. The primary focus on aspects such as caries excavation, isolation techniques, matrix selection, adhesive protocols, light polymerization, and polishing has yielded actionable recommendations based on current expert consensus and supported by the evidence available to date.

However, several limitations must be noted. A significant limitation of the guideline lies in the lack of a formal evaluation of the quality of evidence for each recommendation due to time and feasibility constraints. Consequently, the recommendations primarily rely on consensus, potentially limiting their precision and applicability. Furthermore, the available evidence is sparse or inconsistent for certain aspects of the composite manufacturing process, particularly matrix technique and finishing/polishing. This lack of robust data restricts the ability to provide more definitive guidance in these areas, emphasizing the need for future research to address these gaps and reinforce the evidence base for dental restoration practices.

Regarding the correct processing of composite restorations, both selective and non-selective caries removal methods were shown to be effective. However, selective caries removal appears to offer better outcomes for maintaining pulp health in deep lesions. A systematic review^27^ found no difference in restoration success over two years between selective and two-step caries removal, but it did note the superiority of the selective approach in terms of clinical pulp sensitivity. Additionally, a further meta-analysis^32^ comparing selective and non-selective caries removal revealed no significant difference in the risk of pulp symptoms but a reduction in pulp openings with the selective method. Other reviews^4,52,54^ also supported the decreased risk of pulpal exposure and symptoms associated with selective or staged caries removal, especially in lesions close to the pulp. Lastly, a Cochrane review^55^ concluded that selective or staged removal of carious tissue in deep lesions is more effective than non-selective methods, although the quality of evidence for most comparisons was rated as low to very low.

Most reviews on different types of work field isolation reported no differences between the clinical performance of restorations isolated with rubber dams or cotton rolls,^6,9,18,35,46,47,57^ while some found better results for the use of rubber dam.^39,60^ Little evidence was available on the choice of matrix type *in vivo*, reporting that both metal and acrylic matrices can be used for sufficient proximal contact design.^12,20,21,45^ However, *in vitro* research suggests better proximal contact strength, less marginal excess and more stable marginal ridges with sectional matrices in Class II restorations.^36–38,48,49^

The evaluation of the available literature on adhesive systems lead to limited findings. In older studies, two-step-self-etch and three-step-etch-and-rinse adhesive systems showed slight advantages in the durability and secondary caries resistance of composite restorations. Universal adhesives showed similar results. In contrast, phosphoric acid etching showed clear advantages in the evaluation of enamel adhesion, as it at least reduced marginal discoloration.^1,19,31,39,44,53,58^

Evidence on light polymerization and polishing was poor, which is why the consensus-based statements were based more on standardized protocols and standard clinical practice and less on standardized clinical studies.

In conclusion, the guideline recognizes both selective and non-selective caries excavation methods, with a preference for one-stage selective removal in dentin lesions near the pulp. For work field isolation, both relative and absolute isolation techniques are considered effective, with the use of a rubber dam offering potential long-term advantages. Anatomically preformed sectional matrices are advised for Class II proximal contacts. Using phosphoric acid for etching can enhance enamel margin quality and prevent discoloration. The recommended adhesives are two-step-self-etch, three-step-etch-and-rinse-systems, or universal adhesives. Correct light polymerization, taking into account handling, energy application, and composite shade, is crucial. Bulk-fill composites are deemed safe for up to a 4 mm depth. Finally, polishing composite restorations is recommended to improve surface finish and reduce plaque accumulation.

### Clinical Relevance Statement

This guideline provides practical recommendations for the manufacturing process of composite restorations, outlining caries removal, working field isolation, matrix and adhesive techniques, light curing, and polishing to ensure restoration quality.

## Appendix Part 2

**Table A1:** MEDLINE search term via OVID for the PICO questions

PICO question #6	PICO question #7	PICO question #8	PICO question #9	PICO question #10	PICO question #11
exp Tooth Diseases/ exp Dental Caries/ caries.mp. dental caries.mp. carious lesion*.mp. tooth Decay.mp. dental Cavit*.mp. Cavit*.mp. demineralization*.mp. 1 or 2 or 3 or 4 or 5 or 6 or 7 or 8 or Dental Cavity Preparation/ caries excavat*.mp. caries remov*.mp. residual caries.mp. 11 or 12 or 13 or 14 Randomized Controlled Trials as Topic/ exp Controlled Clinical Trial/ RCT*.mp. randomized controlled Trial*.mp. randomised controlled Trial*.mp. systematic review*.mp. meta Analysis.mp. controlled clinical Trial.mp. randomized.mp. randomised.mp. controlled clinical Trial*.mp. cct*.mp. 16 or 17 or 18 or 19 or 20 or 21 or 22 or 23 or 24 or 25 or 26 or 27 10 and 15 and 28 limit 29 to (yr=”1990 -Current” and (english or french or german or russian))	exp dental restoration failure/ or exp dental restoration, permanent/ or exp dental restoration repair/ or dental marginal adaptation/ or exp diagnosis, oral/ exp Composite Resins/ dental restoration*.mp. filling*.mp. restoration*.mp. composit*.mp. 1 or 2 or 3 or 4 or 5 or 6 Rubber dams/ ((rubber adj dam*) or (oral adj dam*) or (dental adj dam*) or (latex adj dam*) or Kofferdam).mp. (“Optra Dam” or “OptraDam Plus” or OptiDam or FlexiDam or “Hygenic Fiesta”).mp. operatory field isolation.mp. 8 or 9 or 10 or 11 Randomized Controlled Trials as Topic/ exp Controlled Clinical Trial/ RCT*.mp. randomized controlled Trial*.mp. randomised controlled Trial*.mp. systematic review*.mp. meta Analysis.mp. controlled clinical Trial.mp. randomized.mp. randomised.mp. controlled clinical Trial*.mp. cct*.mp. 13 or 14 or 15 or 16 or 17 or 18 or 19 or 20 or 21 or 22 or 23 or 24 7 and 12 and 25 limit 26 to (yr=”1990 -Current” and (english or french or german or russian))	exp dental restoration failure/ or exp dental restoration, permanent/ or exp dental restoration repair/ or dental marginal adaptation/ or exp diagnosis, oral/ dental restoration*.mp. exp Composite Resins/ filling*.mp. restoration*.mp. composit*.mp. 1 or 2 or 3 or 4 or 5 or 6 (matrix adj1 system*).mp. (matrix adj1 band*).mp. matrice*.mp. (separation adj1 ring*).mp. (proximal adj1 contact*).mp. 8 or 9 or 10 or 11 or 12 exp dentistry/ 7 and 13 and 14 limit 15 to (yr=”1990-Current” and (english or french or german or russian))	exp dental restoration failure/ or exp dental restoration, permanent/ or exp dental restoration repair/ or dental marginal adaptation/ or exp diagnosis, oral/ exp Composite Resins dental restoration*.mp filling*.mp. restoration*.mp. composit*.mp. 1 or 2 or 3 or 4 or 5 or 6 Adhesives/ or Dentin-Bonding Agents/ Dental Bonding/ Acid Etching Dental/ Dental Etching/ bonding.mp. (adhes* adj1 system*).mp. adhesive.mp. 8 or 9 or 10 or 11 or 12 or 13 or 14 Randomized Controlled Trials as Topic/ exp Controlled Clinical Trial/ RCT*.mp. randomized controlled Trial*.mp. randomised controlled Trial*.mp. systematic review*.mp. meta Analysis.mp. controlled clinical Trial.mp. randomized.mp. randomised.mp. controlled clinical Trial*.mp. cct*.mp. 16 or 17 or 18 or 19 or 20 or 21 or 22 or 23 or 24 or 25 or 26 or 27 7 and 15 and 28 limit 29 to (yr=”1990 -Current” and (english or french or german or russian))	exp dental restoration failure/ or exp dental restoration, permanent/ or exp dental restoration repair/ or dental marginal adaptation/ or exp diagnosis, oral/ exp Composite Resins/ dental restoration*.mp. filling*.mp. restoration*.mp. composit*.mp. 1 or 2 or 3 or 4 or 5 or 6 Curing Light, Dental/ or “Light-Curing of Dental Adhesives”/ or Polymerization/ light cur*.mp. polymeri*ation.mp. curing protocol.mp. 3s PowerCure.mp. dual cur*.mp. 8 or 9 or 10 or 11 or 12 or 13 Randomized Controlled Trials as Topic/ exp Controlled Clinical Trial/ RCT*.mp. randomized controlled Trial*.mp. randomised controlled Trial*.mp. systematic review*.mp. meta Analysis.mp. controlled clinical Trial.mp. randomized.mp. randomised.mp. controlled clinical Trial*.mp. cct*.mp. 15 or 16 or 17 or 18 or 19 or 20 or 21 or 22 or 23 or 24 or 25 or 26 7 and 14 and 27 limit 28 to (yr=”1990 -Current” and (english or french or german or russian))	exp dental restoration failure/ or exp dental restoration, permanent/ or exp dental restoration repair/ or dental marginal adaptation/ exp Composite Resins/ dental restoration*.mp. filling*.mp. restoration*.mp. composit*.mp. 1 or 2 or 3 or 4 or 5 or 6 finishing.mp. polishing.mp. contouring.mp. 8 or 9 or 10 Randomized Controlled Trials as Topic/ exp Controlled Clinical Trial/ RCT*.mp. randomi*ed controlled Trial*.mp. systematic review*.mp. meta Analysis.mp. controlled clinical Trial.mp. randomi*ed.mp. controlled clinical Trial*.mp. cct*.mp. 12 or 13 or 14 or 15 or 16 or 17 or 18 or 19 or 20 or 21 7 and 11 and 22 limit 23 to (yr=”1990 -Current” and (english or french or german or russian))

**Table A2:** Excluded publications with reasons

PICO question	Publication	Reason for exclusion
6	Browning 20158	Summary of Bjorndal et al., 2010
	Clarkson 202112	Only study protocol
	Fontana 201422	Summary of Schwendicke et al., 2013
	Giacaman 201823	Non-systematic review
	Hamama 201525	No survival analysis
	Jacobsen 201128	Follow-up insufficient
7	De Lourdes Rodrigues 200615	Follow-up insufficient
	Pignoly 199043	Commentary
	Rau 200644	Follow-up insufficient
8	Andersson-Wenckert 20021	Follow-up insufficient
	Anonymous 20142	Commentary
	Arhun 20133	*In vitro*
	Belvedere 19945	*In vitro*
	Belvedere 20064	Commentary
	Browning 20007	Commentary
	Burke 20019	Commentary
	Cenci 200610	Follow-up insufficient
	Cho 201011	Commentary
	Cvitko 199214	*In vitro*
	Derrick 200016	Commentary
	Din 199217	Commentary
	Doukoudakis 199618	Commentary
	Durr 201819	Follow-up insufficient
	Gomes 201524	Follow-up insufficient
	Kaplowitz 199731	Commentary
	Kwon 201433	*In vitro*
	Loomans 200637	Follow-up insufficient
	Loomans 200736	Follow-up insufficient
	Owens 201642	Commentary
	Rosin 200748	No different matrix designs were evaluated
	Rosin 200349	No different matrix designs were evaluated
	Van der Vyver 200253	Commentary
	Wirsching 201155	Follow-up insufficient
9	Coe 201713	Summary of Schroeder 2017
	Farsai 201821	Summary of da Silva 2018
	Leloup 200134	*In vitro*
	Lima 202135	*In vitro*
	Madrid troconis 201739	*In vitro*
	Rice 201545	No survival analysis
	Rocha 201847	Wrong intervention
	Sia 201850	Summary of da Silva 2018
	Zhang 2020a56	*In vitro*
	Zhang 2020b57	Wrong intervention
10	Braga 20056	Non-systematic review
	Cvitko 199214	*In vitro*
	Hardan 200926	*In vitro*
	Kays 199132	*In vitro*
	Sea ice 201840	*In vitro*
	Munchow 201841	*In vitro*
	Rice 201746	*In vitro*
	Strassler 201851	Commentary
11	Dutra 201820	*In vitro*
	Hellak 201527	Wrong topic
	Jaramillo-Cartagena 202129	*In vitro*
	Young 200530	Insufficient follow-up
	Lussi 199238	Wrong topic
	Teixeira 201952	Insufficient follow-up
	Wakefield 201354	Commentary
